# Derma rollers in therapy: the transition from cosmetics to transdermal drug delivery

**DOI:** 10.1007/s10544-020-00530-3

**Published:** 2020-10-26

**Authors:** Leonna Dsouza, Vivek M. Ghate, Shaila A. Lewis

**Affiliations:** Department of Pharmaceutics, Manipal College of Pharmaceutical Sciences, Manipal Academy of Higher Education (MAHE), Manipal, Karnataka 576104 India

**Keywords:** Derma rollers, Micro-needling, Cosmetics, Transdermal drug delivery, Skin, Micropores

## Abstract

Derma roller, a device rolled onto the skin to form micropores, is extensively used for cosmetic purposes. The pores thus created are utilized to either result in the induction of collagen production, leading to glowing and wrinkle-free skin or for permeating the applied formulations to the site of action within the skin. Recent studies have shown the benefits of using derma rollers for transdermal delivery of drugs. In the nascent stage, this approach paves a way to successfully breach the stratum corneum and aid in the movement of medications directed towards the dermis and the hair follicles. The review essentially summarizes the evidence of the use of derma rollers in cosmetic setup, their designing, and the preclinical and clinical reports of efficacy, safety, and concerns when translated for pharmaceutical purposes and transdermal drug delivery.

## Introduction

Microneedling, a term familiar to most dermatologists today, has continuously evolved since its inception in the early twentieth century. The technique of microneedling is built upon non-pathogenic puncturing of the skin with micro-sized needles, thereby stimulating the underlying cells to increase the production of growth factors and the vital dermal ingredient, collagen (Aust et al. 2008; Hou et al. 2017). The use of microneedles is painless relative to the conventional hypodermic needles since they cannot penetrate the dermis layer (Azmana et al., 2020). Using microneedles also reduces trypanophobia (needle phobia) related to the use of hypodermic needles for parenteral delivery (Jamaledin et al., 2020). Dermatologists accept significant microneedling applications in the antiaging and reversal of wrinkles, and as a rejuvenating therapy for smooth and youthful-looking skin (Alster and Graham 2018; Ramaut et al. 2018; Singh and Yadav 2016). As a cosmetic treatment option, microneedling also overcomes the scars caused by injury to the skin resulting from acne, surgery, keloids, and stretch marks, and the most prominent and difficult to counter problems of hair loss and regrowth. Though there exist non-invasive techniques to address the above cosmetic conditions, including dermabrasion, chemical peeling, and laser therapy, they are inherent with a high risk of depigmentation, additional scarring of the skin, and the undesired clinical outcomes (Chandrashekar et al. 2014).

**Fig. 1:**
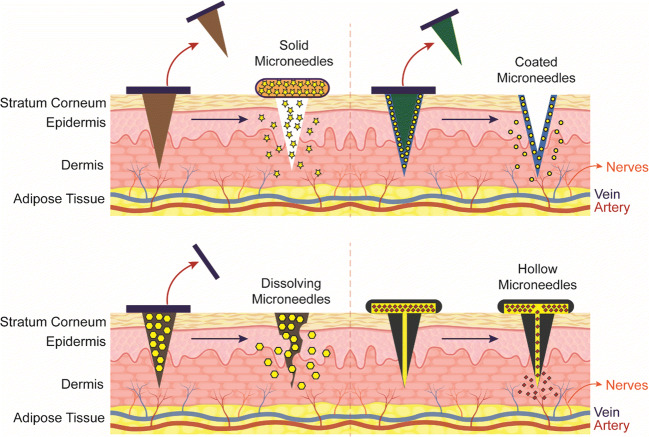
Representation of different types of microneedles including solid, coated, dissolving and hollow microneedles

The limitation such as needle injury, phobia, need of specially qualified personnel, which will also raise the delivery costs posed by traditional dosage forms such as intradermal and intravenous injections, can be surpassed using microneedles. The technology was examined to deliver not only small molecules but also complex macromolecules, cosmeceuticals, and micro/nanoparticles. At present, microneedle applications have grown beyond their biomedical applications, including long-term treatment of diseases, immunobiological management, diagnosis of diseases, and the cosmetic industry. Microneedles can also be used to distribute large amounts of macromolecules such as insulin, immunobiological products, proteins, and peptides in a controllable way, which can be passed directly into the epidermis to enhance their therapeutic potency for long term treatment (Yang et al. 2019). Microneedles are primarily made from polymers as a specialized form of transdermal drug delivery to encapsulate and control the release of drugs. However, other microneedles were manufactured from metals, glass, silicon, ceramics, maltose, or carbon (Nagarkar et al. 2020). The microneedles (Fig. 1) are categorized according to their function: solid, coated, hollow, dissolving microneedle (Ahmed Saeed AL-Japairai et al. 2020), and liquid injection system (Mir et al. 2020).

### Solid microneedles

As they are inserted and removed to form micron-scaled pores on the skin surface, they can be used as a skin pretreatment—the microchannels created to act on a ‘poke and patch’ principle. A modification of a traditional reliable microneedle technique is the ‘scrape and repair’ process by which micro projections are scraped across the skin to create micro-abrasions. The drug solution is spread on specific micro projections inside a patch. Microneedle patch contains an array of solid microneedles that produce a grid of micropores through which medications may be delivered to the skin for local or systemic drug absorption (Wermeling et al. 2008). Another variation is using a roller containing microneedles, piercing the stratum corneum as the roller spins on the skin several times. Based on this definition, commercially available Derma-rollers are used for skin pore opening procedures.

### Coated microneedles

These microneedles operating on the concept of ‘coat and poke’ contain a layer of solid microneedles that have a coat containing drug solutions. The most popular approach is dip coating, but it is complicated because of precise control to ensure that the microneedles are adequately injected into the dipping solution.

### Hollow microneedle

It is a small variant of the traditional hypodermic needles. Drug delivery is accomplished by the flow of liquid formulation, which is driven by pressure. Substantial doses can be distributed via this technique into the dermal layer. Hollow microneedles are challenging to manufacture because of their composition and fragility (Ita 2015).

### Dissolving microneedles

They work on the concept of ‘poke and release.’ Compared to other microneedles, they are easy to fabricate by micro-molding methodology and use and have gained considerable popularity in recent years. Dissolving microneedles are manufactured from biodegradable materials such as sugars or polymers, biodegradable, and filled with therapeutic agents. Upon application, the microneedles dissolve into the skin and release the therapeutic agent(Yang et al. 2019).

### Microneedle liquid injection system

It is used to supply the necessary drug concentrations to chronic wounds. Microneedle liquid injection devices not only can provide quick, precise and painless delivery to chronic wounds but can also achieve additional advantages compared to hypodermic needles (Mir et al. 2020)(Waghule et al. 2019).

The microneedling was first developed and patented in 1976 for transdermal delivery (Gerstel and Place, 1976). The microelectronics industry has made significant progress since 1990, which has immensely benefited the microfabrication of microneedles (Prausnitz et al. 2004). Before the introduction of microneedling for cosmetic use, the practice called ‘subcision’ was developed wherein the cosmetic surgery was achieved by using a tri-beveled hypodermic needle (ORENTREICH and ORENTREICH 1995; Ramaut et al. 2018; Thi Minh et al. 2019). Equally effective during those days, subcision resulted in a painful and non-uniform action and required the insertions at various regions of the skin to be made wrinkle and scar-free. Subcision worked by breaking down the aggregated strands of fibrotic tissues, followed by a deposition of new collagen due to the healing of the minor wounds. Moving ahead of conventional microneedling, derma rollers can uniformly and painlessly stimulate the underlying areas of the skin, allowing a more profound rejuvenation and redevelopment of collagen (Lee and Hong 2008).

## Transition of derma roller from cosmetic to transdermal delivery

**Fig. 2:**
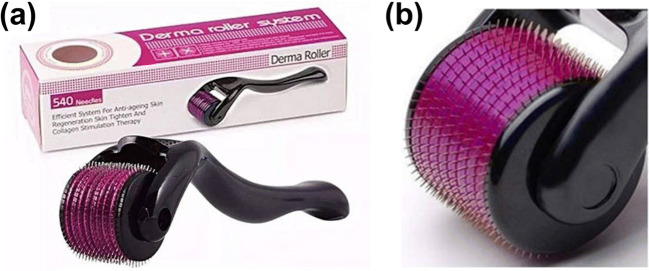
Representative image of a typical dermaroller (**a**) and the array of needles (**b**) taken from the Derma Roller System (D.R.S.), a needle length of 0.5 mm, a total of 540 needles. The images presented in the figure have been taken from the manufacturer’s websites for representation purposes

Skin is the most extensively visible portion of the human body and is cross-sectionally divided into the epidermis (approximate thickness of 0.03 to 1 mm); the dermis (approximate thickness of 0.7 to 4 mm), and the hypodermis. Several layers of dead cells line the outer surface of the epidermis exposed to the external air and environment, collectively called the ‘stratum corneum’ which is regarded as the primary barrier of the body (Henry et al. 1998; Ita 2015,(Donnelly et al. 1965). Solute transport through this layer is mainly through passive diffusion, and no active transport processes have been reported (Brown et al. 2008). The numbers of cosmetic antiaging formulations were limited in their activity due to the improper permeation through the stratum corneum as the wrinkles occur deep within the dermis. Derma rollers (Fig. 2) were thus introduced to establish a pathway for the antiaging formulations to reach the site of action and exert their effect. Camirand and colleagues (1997) first observed that needle abrasion was responsible for the healing of the scar tissues from their experiment of using an empty tattoo gun on surgical scars(Andre Camirand 1997). Following the lead, Fernandes et al. (2002) developed the technique of percutaneous collagen induction wherein the treatment involved the partial destruction of the epidermis using a needle drum. The study conducted on 480 patients concluded with a 60 to 80% improvement in collagen and elastin fibers and reiterating that derma rollers could serve as an alternative to non-invasive therapy of stimulating the formation of collagen while maintaining the overall structure of the epidermis (Fernandes 2002).

**Fig. 3:**
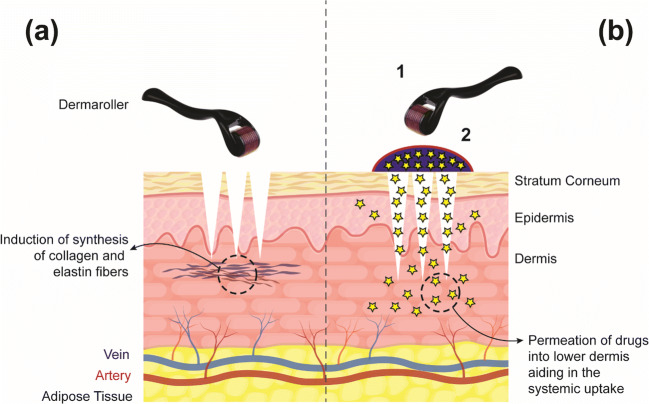
Overview of the application of derma rollers for (**a**) cosmetic and (**b**) transdermal application. In the case of transdermal delivery, the skin is pretreated with the dermaroller (1) followed by use of the drug formulation (2)

Taking the lead from successful derma roller cosmetic therapy, researchers directed the applications of derma rollers in the development of transdermal drug delivery systems (Fig. 3**)**. Although the success of the transdermal application of derma roller applications is minimal, numerous examples merit the need for research in the area of systemic drug delivery. Ahmed and colleagues pointed out that pretreatment of healthy skin with derma rollers can increase the penetration of the drug-loaded liposomal formulations more than two folds compared to the passive delivery (Ahmed et al. 2019). Majorly derma rollers have been exploited in combination with physical methods such as iontophoresis and ultrasound exposure resulting in improved subcutaneous drug delivery, efficacy and compliance, and reduced toxicity (Abbasi and Wang 2019). Bahadoran and team (2015) suggested that microneedling, in addition to the ablative fractional carbon laser, significantly enhanced the delivery of aminolevulinate, currently used in photodynamic therapy (Bahadoran et al. 2015). Well-suited for the delivery of small molecular weight compounds, pretreatment with derma rollers can also enable the permeation of a large dose of the drug when the active ingredient is left for a longer time on the skin in the form of polymeric patches (Wermeling et al. 2008).

### Cosmetic use of derma rollers

Majorly exploited for aesthetic purposes, derma rollers are available in various forms and designs. A simple drum-shaped derma roller with a total of eight rows bearing 192 fine needles was used for the acne scars treatment (Pahwa et al. 2012). The needles had a length in the range of 0.5 to 2.0 mm and an effective diameter of 0.1 mm. The needles were produced on silicon or medical grade stainless steel by reactive ion etching methods. Derma roller in cosmetic application intends to boost the efficacy of skincare products by permeating the stratum corneum, thereby reaching the lower tissues of the skin (Table 1). These Derma Rollers have a curved structure that is simple to maintain and use (Lee and Hong 2008). The handle is produced of colored plastic, and needles made of titanium alloy are held in the roller head. These needles are tough and durable, making this derma roller one of the long-lasting beauty instruments well suited for needling big and small regions. Valid on all skin types, derma roller is perfect for stretch mark removal and treatments for scar decrease on any portion of the body. Titanium needles will guarantee a longer shelf-life with minimal requirement of replacing the needles. Miniature derma roller variants have been created called derma stamps. The derma-stamp operation can be carried out even quicker than the roller (Bahuguna 2013).

**Table 1:** Needle dimensions and the reported use of derma rollers for the administration of cosmetic formulations

Drug/Formulation	Length of derma roller (mm)	Reference
Fusidic acid	1.5	(Thi Minh et al. 2019)
Retinoic acid, Ascorbic acid	3.0	(Fernandes and Signorini 2008)
Rucinol and sophora alpha	0.5	(Fabbrocini et al. 2011)
Anti-aging serum	0.25	(Kassir and Sahni 2013)
Tazarotene gel	1.5	(Afra et al. 2019)
Melanocyte/keratinocyte suspension	0.5, 1.0	(Palareti et al. 2016)
Glycerol	0.5	(Yoon et al. 2008)
Glycolic acid	1.5	(Sharad 2011)
Methylaminolevulinate	0.2	(Bahadoran et al. 2015)
5-aminolevulinic acid (A.L.A.) and methyl aminolevulinate (M.A.L.) is	0.3, 0.6	(Mikolajewska et al. 2010)

**Fig. 4:**
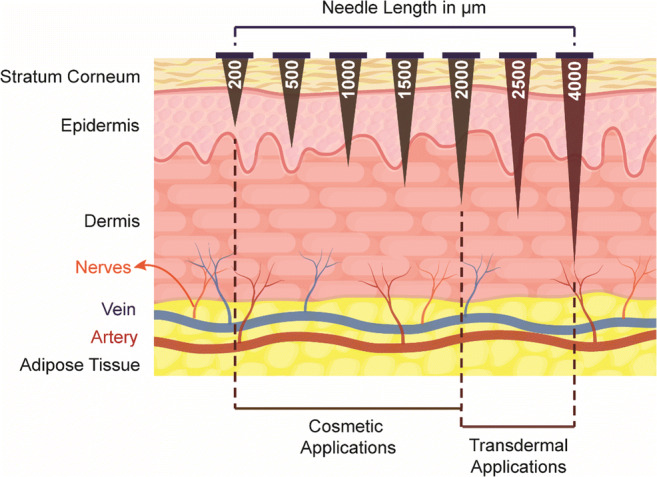
Variations of the needle length in derma rollers and their application

When non-fibrotic, healthy skin is derma rolled, healing responses are stimulated only by T.G.F. β3 regulated collagen induction, by degrading the scar tissue (Busch et al. 2018). Derma rollers create pores in the dermis and begin a complex chemical sequence automatically. The platelets initiate the release of different factors, which cause a chain reaction, eventually releasing other growth factors. Fibroblasts shift into the region, and this increase in activity invariably leads to even more collagen and elastin production (Fernandes and Signorini 2008). It leads to the cosmetic use of the derma roller. A comparison of different needle lengths and possible layer up to which the micropores may reach is provided in Fig. 4***.***

#### Derma stamp

**Fig. 5:**
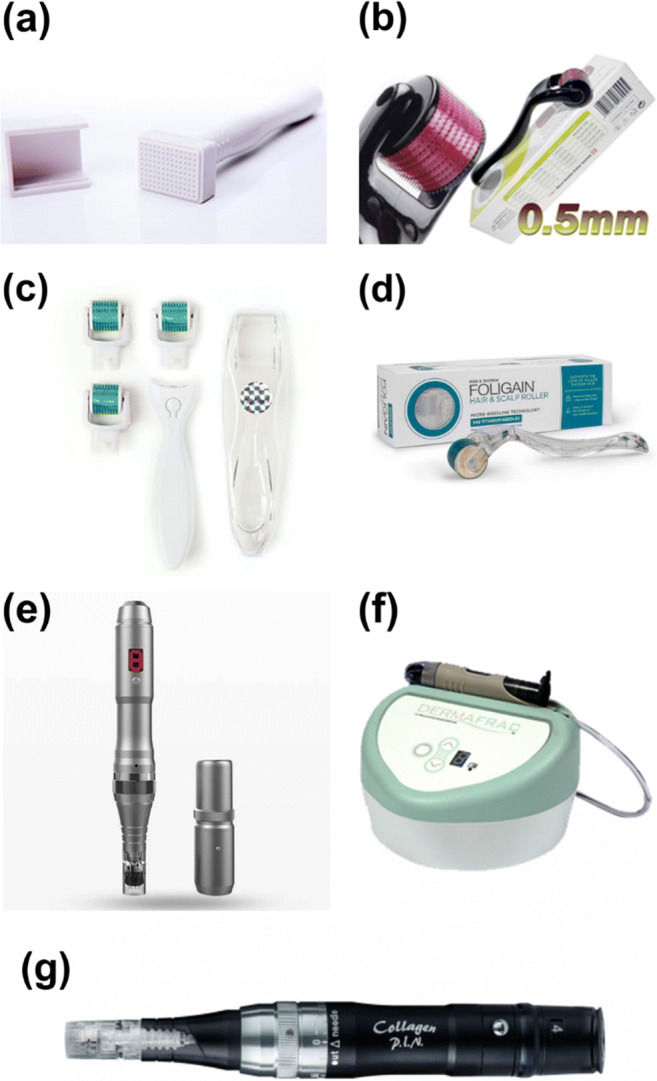
Different types of dermarollers used for cosmetic purpose; (**a**) Derma stamp; (**b**) Homecare dermaroller; (**c**) Automated dermaroller; (**d**) Scalp roller; (**e**) Derma pen; (**f**) Dermafrac; and (**g**) Collagen P.I.N.. The images presented in the figure have been taken from the manufacturer’s websites for graphical representation

In contrast to the parent derma rollers, usually characterized by several needles resting on a barrel head, the derma stamp is characterized by few needles resting on a flat head. Considered as a miniature version of the derma roller, the derma stamp has 2 mm long needles of 0.1 mm in diameter (Doddaballapur 2009) (Fig. 5a). Derma stamps are meant for the treatment of specific portions of the skin, such as the scars of varicella and postaumatic injuries. The needle length of this derma roller varies from 0.2 mm to 3 mm with a diameter of 0.12 mm and can be more useful when working with particular areas repeatedly. Derma stamp can be used to treat the individual scars in a focused manner when compared to the conventional derma roller. Derma stamp creates vertical penetrations in the skin and is deemed suitable for separate scars and wrinkles (Singh and Yadav 2016).

#### Homecare derma rollers

Derma rollers meant for homecare are less than 0.15 mm long. They are mainly used for the dermal supply of drugs such as lipopeptides and other antiaging products (Fig. 5b). These derma rollers (C-8) have a needle length of 0.15 mm and are self-useable for the patients and are accessible for pore size reduction, reduction of fine lines, and decreasing sebum manufacturing. They are also used for the delivery of antiaging and lipoproteins. They can be used up to 100 times, twice or three times a week. The rollers should be washed and shaken dry in hot tap water after use. Another authorized device for home use is the BeautyMouse. This device has three different drums on which 480 needles are arranged with a needle size of 0.2 mm. It was created to guarantee the coverage of more prominent regions of the skin, like upper limbs, hindquarters, for the stretch mark therapy of the stomach or thigh and cellulite (Mccrudden et al. 2015).

#### Automated dermarollers

A battery with disposable heads powers automated rollers, which can be used in more than one patient as disposable heads only need to be altered (Fig. 5c). Pressure on scars is uniform than any other type of hand-held derma roller. In the automated roller, the needle cartridge after single use can be replaced by different needles. Here the operator can adjust the frequency and penetration depth of the needle. The automated roller used currently has a needle length of range 0.5 to 1.5 mm (Ablon 2018).

#### Scalp roller

Used to treat thinning hair, the scalp roller utilizes titanium needles, unlike steel needles used in other derma rollers (Fig. 5d) (Deepak and Shwetha 2014). The micro-needling procedure is thought to function best for people with androgenic alopecia**.** Microneedling, in addition to the existing patient’s treatment strategy, has positive benefits, and new hair formation will commence after 8–10 sessions (Dhurat and Mathapati 2015).

#### Dermapen

Dermapen is an automated tool used for fractional mechanical resurfacing (Fig. 5e). The device is ergonomic and used as a guide to adjust the needle length and is provided with a disposable needle. The tip is arranged in rows with 9–12 needles. This device functions in a vibrating manner in two modes, one at a high rate (700 cycles/min) and another at a low rate (412 cycles/min) (Iriarte et al. 2017). Dermapen is packed with the benefit of being used several times by different patients as it has disposable needles, needle tips are also concealed within the manual. They are comfortable to treat delicate regions like the around the eyes, lips, and nose without damaging the skin around. It makes the operation less painful and more cost-effective so that it can be purchased as a new tool every time. The dermapen technology solves pressure variation implementation and subsequent in-depth penetration (Singh and Yadav 2016). The currently available marketed dermapen is called the Eclipse Micropen™.

#### Collagen P.I.N

Collagen P.I.N. is a mechanical microneedling tool with a sterile needle top comprising 12 small microneedles (Fig. 5f). It is ideal for elastin and collagen stimulation. It can work on fine lines, imperfections, skin texture, and reduce acne marks (Puiu et al. 2018).

#### Dermafrac

Dermafrac is the alteration of the microneedling method using micro conduits produced by microneedling to transmit antiaging growth factors or other pharmaceutical-property topical agents passively (Fig. 5g). DermaFrac therapy incorporates micro channeling with the accurately calibrated needle piercing with vacuum-assisted infusion simultaneously (Kim et al. 2012).

### Transdermal use of derma roller

As the medical use of microneedling is now expanding beyond scar treatment since the original derma roller was implemented, numerous modifications and developments have been made (Ryan 2018). A 100,000 times increase in the flux of the drugs is claimed following the derma roller treatment (Barry 2001). Derma roller results in disruption of the stratum corneum cells, which weakens the main barrier of the skin, causing an increase in the absorption of applied compounds (Table 2) (Brown et al. 2008). Derma rollers were demonstrated to perforate through the skin into the epidermis through the stratum while avoiding contact with nerve fibers and blood vein primarily in the dermal layer (Haq et al. 2009). The main advantage of using derma rollers is the pain-free delivery of active pharmaceutical ingredients of smaller and larger molecular weights (R.R. Singh et al. 2011).

**Table 2:** Needle dimensions and the reported use of derma rollers for the transdermal administration of pharmaceutical formulations

Drug	Length of dermaroller (mm)	Reference
Acetylsalicylic acid	0.6	(Park et al. 2010)
Invasomal preparation of Carboxyfluorescein and radiolabeled mannitol	0.5, 1.5	(Badran et al. 2009)
DOX/CEL co-loaded liposome	0.5	(Ahmed et al. 2019)
Diclofenac Sodium	0.5	(Patel et al. 2015b)
Insulin	0.25, 0.5, 1.0	(Zhou et al. 2010)
Naltrexone	0.62	(Wermeling et al. 2008)
5-Flurouracil	0.5	(Naguib et al. 2014)
Ondansetron	0.5	(Patel et al. 2015a)
Sumatriptan	0.5, 1.0	(Nalluri et al. 2015)
Ovalbumin	0.2, 0.5, 1.0	(Kumar et al. 2011)
Hydrochloride and amlodipine besylate	0.5	(Kaur et al. 2014)

Derma rollers have been recently studied for their ability to increase the permeation of the pharmaceutical formulation. The flux of salicylic acid across the intact epidermal portion of human skin was increased by 21 fold and 47 fold when microneedle rollers with 100 and 200 needles were used respectively (Park et al. 2010). Derma roller increased the availability of a variety of NSAID drugs like ketoprofen, ibuprofen, diclofenac, and paracetamol. Hence, it was found beneficiary for drugs having low lipophilicity and a high melting point (Stahl et al. 2012). Microneedling establishes more extensive routes of transportation to allow large drugs like insulin to be delivered. It was also shown that an increase in needle length increased the permeation rate of insulin. Having the advantage of easy handling, the derma roller acts as a promising tool for delivering biologically active compounds like proteins (Zhou et al. 2010). In vitro permeation studies by Nalluri et al. (2015) showed that pretreatment with a derma roller could significantly improve the sumatriptan permeation compared to passive permeation (Nalluri et al. 2015). The transcutaneous immunization on the skin region pretreated with nanoparticles of protein antigen showed more response than the use of protein antigen alone (Kumar et al. 2011).

The use of derma roller is usually followed by local antibiotic cream and photoprotection. Donnelly et al. (2009) showed that the microbial entry through the skin pore created by the derma roller is significantly less compared with the skin pore created by the hypodermic needle (Donnelly et al. 2009). They also didn’t find any entry of the microorganism through the orifice formed by the derma roller on the epidermis (Doddaballapur 2009). The key features of derma rollers are self-administration and cost-efficient, combined with their versatility in treating different areas (Patel et al. 2015b). The original microneedle technology has grown to include the direct injection of pharmaceutical formulations, gene therapy, and vaccines to establish transportation routes without pain through the stratum corneum (Sivamani et al. 2007).

The pores created by the derma roller on the skin were used as liquid electrodes, which provided a regular and profound tissue electrical field with flexible integrated electroporation array (FIEA) introduced pulse stimulus (Huang et al. 2018). Derma roller could be a significant advantage in pandemic diseases. There will be a crucial difference in gathering massive patients at convenient locations and getting enough medical personnel to administer medicines and potential other therapies. It would greatly ease this obstacle by requiring minimally trained staff to deliver the medication, and enabling self-administration would improve the efficiency even more (Prausnitz and Langer 2008).

In clinical practice, the actual depth of produced microchannel fit tightly with the selected range of needle lengths of 0.25 mm to 1 mm but does not more frequently fit the chosen range of long needle lengths from 1.5 to 2.5 mm as identified by histological measurements (Sasaki 2017). In the survey conducted among the public and professionals, varying opinions were received (Birchall et al. 2011). Both the groups agreed that derma rolling would make benefactions for children and needle-phobic, whereas the professional group notably claimed that it would help acute and chronic delivery. With a low frequency of adverse effects, skin needling seemed healthy (Harris et al. 2015). In contrast to the derma rollers for cosmetic therapy, the reports available for transdermal applications are very few.

#### CIT-8

**Fig. 6:**
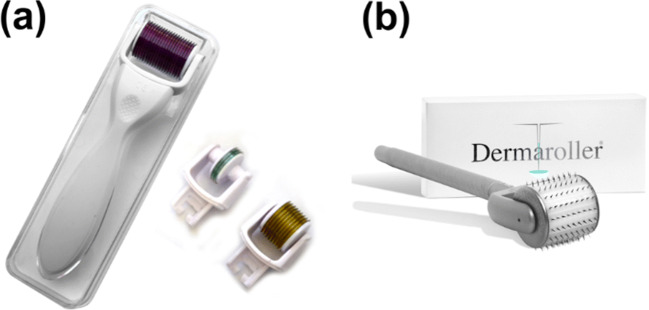
Different types of dermarollers used for transdermal delivery; (**a**) C.I.T. and (**b**) M.F. The images presented in the figure have been taken from the manufacturer’s websites for representation purposes

C.I.T. -8 is a transdermal derma roller used to induce collagen production in the serum with a needle size of 0.5 mm (Fig. 6a) (Mohan et al. 2020). It is made of a 12 cm plastic handle, like a paint roller, having a diameter of 20 mm and length of 20 mm, connected to a cylinder. The cylinder surface contains 24 circular clusters of 8 needles each (together of 192 needles), with 0.02 mm diameter. Along with a unique medically authorized adhesive, needles and disks are firmly bound (Fabbrocini et al. 2011).

#### MF-8

A derma roller with a needle length of 1.5 mm, MF-8 is used to destruct the scar collagen bundle and create deeper pores on the epidermis and dermis (Fig. 6b). The tool has a 12 cm long handle which holds a cylinder at the end, having a diameter of 2 cm and a thickness of 2 cm. The cylinder surface carries 192 stainless steel needles in 8rows. Each needle is 0.25 mm in diameter and 1.5 mm in length. They have a 15-degree and 30- degree radial arrangement concerning the roller core (Waghule et al. 2019).

#### MS-4

A variant of MF-8, MS-4, has a needle length of 1.5 mm and a cylinder of 1 cm in length and diameter used for providing a better penetration and deeper permeation (Singh and Yadav 2016).

## Notable preclinical and clinical evidence

Being heavily used in a dermatological setup, reported clinical evidence are scarce for the effectiveness of derma rollers in cosmetic and therapeutic interventions (Table 3).

**Table 3:** Preclinical and clinical evidence of efficacy of the use of derma rollers

Indication	Patients or animals	Objective	Outcome	Reference
Scars	30 male rats	Evaluation of derma rolling with and without skincare products.	The highest increase in epidermal thickness with four repetitive sessions of micro needling along with topical vitamin A and C.	(Zeitter et al. 2014)
Scars of acne vulgaris	30 patients	To evaluate the effect of derma roller on scaring.	74.1% showed an excellent response.	(Patil and Patil 2016)
Atrophic scars	36 patients	Evaluation of the efficacy of derma roller device for atrophic facial scars.	Good results in 22 patients and excellent response in 4 patients were showed by VAS analysis.	(Dogra et al. 2014)
Atropic acne scars in Vietnamese Patients	31 patients	To evaluate the effectiveness and safety of derma roller in Vietnamese patients	Lipper and Perez score dropped to 36.4+ 12.07 at baseline of 23.16+ 15.01 at final treatment	(Thi Minh et al. 2019)
Melanoma	30 mice	To evaluate the use of derma roller device in combination with DOX and C.E.L. loaded liposomes	Derma roller treatment before gel application improved the tumor inhibition	(Ahmed et al. 2019)
Acne scar	31 patients	To study the safety and efficacy of microneedling fraction radiofrequency	Goodman and Baron’s Global Acne Scarring system shoed excellent improvement	(Chandrashekar et al. 2014)

### Evidence related to cosmetic use

Schwar and Laaff (2011) conducted a clinical study with eleven patients exhibiting posttraumatic scars (Schwarz and Laaff 2011). The Caucasian patients were aged from 22 to 51 years, including five males and six females. In the study, the MF-8 type of derma roller was used. Before the beginning of the treatment with the derma roller, a punch biopsy was conducted on the side of the face on which the surgery was to be done. Microneedling was performed in a criss-cross way under local anesthesia, which resulted in a consistent pattern of punctures and micro bleeding. After eight weeks, another punch biopsy was taken in the treated region 2 cm from the first region. Histological characteristics of the samples were evaluated in a blind study by the independent dermatologist and pathologist. They assessed for total collagen, epidermal thickness, and elastic fibers. The biopsy specimens on histological examination showed a noticeable rise in the elastic fibers. Among eleven patients, seven of them showed a significant increase in the elastic fibers. The increase in elastic fibers in these patients was 1 to 2 fold compared to the normal, and one of the patients showed a tenfold increase in elastic fibers.

Dogra et al. (2014) conducted a clinical study with 36 adult patients suffering from severe to moderate facial scarring due to acne (Dogra et al. 2014). The study included 26 females and ten males within the age group of 18 to 40 years. The derma roller used was a cylinder-shaped roller attached to the handle. It had 192 stainless steel microneedles covering the cylinder in 8 rows. The needles were 1.5 mm long, with a diameter of 0.5 mm. The microneedling therapy was carried out in five sittings, at an interval of once per month. The average acne scar evaluation score for 30 patients was 11.3 + 3.12. The average acne scar evaluation score dropped to 6.5 + 2.71 after one month since the last sitting. The distinction was statistically significant from the baseline. The mean acne scar evaluation score difference in men and women between baseline and end of research was 5.88 + 1.73 and 5.00 + 1.57, respectively. As per the investigators, 50–70% improvement was found by the majority, and all the patients had experienced more than 20% of improvement. In 40% of patients, side effects were observed, and in the majority of the patients, they were mild.

### Evidence related to transdermal use

Ahmed et al. (2019) reported the transdermal efficacy of derma rollers in four-year-old naked BALB/C mice (Ahmed et al. 2019). B16 murine melanoma cells were injected subcutaneously to cause skin tumors. Derma roller with 540 needles having a length of 500 μm and a diameter of 50 μm was used for the treatment. The study constituted various treatments of doxorubicin liposomal gel, microneedling plus the liposomal gel, a combination of doxorubicin and celecoxib liposomal gel, and microneedling plus the doxorubicin and celecoxib liposomal gel, in addition to the untreated control group. The gel (200 mg) containing doxorubicin (2 mg) with and without celecoxib (10 mg) was applied to the experimental animals following the derma roller therapy for ten times with a pressure of 50 g. As the tumor grew to a volume of 10 mm^3^, the liposomal gel was applied to the skin area where the cancer was induced. The control group showed rapid tumor growths were as the drug formulations showed efficient tumor growth inhibition at different degrees. After consecutive eight days of treatment, the tumor volume reduction showed that pretreatment with the derma roller before applying the gel showed a significant increase in the drug permeation. Micro pores created by the derma roller increased the skin permeability of liposomal drug loaded formulation, thereby showing an enhanced antitumor effect. The weight of the tumor of the derma roller treated group before application of the gel was the smallest in comparison to the untreated group. The pretreatment of mice with derma roller and then with the gel resulted in the lower and loosely distributed melanoma cells compared with the mice, which were only treated with the gel without prior treatment with the derma roller.

The clinical study conducted by Fabbrocini et al. (2011) justified using derma rollers for transdermal application (Fabbrocini et al. 2011). The study included 20 female patients with melasma falling within the age range of 32 to 60 years. The Derma roller of model C.I.T. 8 was used in this study, which consisted of the plastic handle, which is 12 cm long, which is attached to a cylinder that has 20 mm of diameter and length. Homecare derma roller eight was also used with 196 needles arranged in a row of 8, a length of 0.13 + 0.02 mm. Melasma was evaluated by the melasma area and severity index (MASI). The first therapy was carried out by another dermatologist.in which derma roller was rolled over melasma areas vertically, horizontally, and diagonally. Treatment was repeated twice a month with an interval of 1 mo. As the first treatment ended, each patient was taught to use derma roller C8. This device does not require any anesthetic application, and also it doesn’t cause any bleeding. They were informed to apply the depigmenting serum on the right hemifacial areas of melasma. Patients were asked to do this every day for 2 mo. Results were analyzed after the completion of the two-session of study. As per the detailed evaluation, it was found that the treated areas with a combination of derma roller and depigmenting serum showed better results compared to the use of only depigmenting serum. The combination therapy had a baseline MASI mean score of 19.1. It decreased to 14.4 on the first treatment and further decreased to 9.2 on the second treatment. The application of the depigmenting agent alone had a baseline score of 20.4, which dropped to 17.4 on the first treatment and also reduced to 13.3 on the second treatment.

### Safety and concerns

Most of the cosmetic procedures available today pose a particular risk to the patient seeking treatment. Along similar lines, microneedling or therapy with derma rollers are not entirely free of side-effects and contraindications (Sharad 2011; Soltani-Arabshahi et al. 2014; Zeitter et al. 2014). These undesired effects are common to both cosmetic and transdermal systems developed with the derma rollers, excluding the contributions from the drug formulations used during the therapy. The most commonly observed effect is skin irritation, mild bleeding, peeling, and rashes (Badran et al. 2009; Donnelly et al. 2009). Certain cases, these immediate effects can also be accompanied by infections (Ryan 2018). Particularly in the cases where the patient is pregnant, have evidence of skin infections and diseases such as psoriasis and eczema, and open wounds, microneedling, or the use of a derma roller may exaggerate the conditions. Though micropores production and healing take place very quickly, and the procedure being considerably non-invasive, it can be the most preferred option for cosmetic and skin rejuvenation. To date, the needles have not been associated with any adverse effects as the materials used in the fabrication are routinely used in a dermatological setup. Like all needle containing devices, it is of utmost importance that the user refrains from sharing the derma roller with others, preventing the transfer of undesired health problems.

### Advantages of dermaroller compared to microneedle patch

Although the dermaroller and microneedle patchwork on the same principle, dermaroller can be advantageous over the microneedle patch. Dissolving microneedles patch being water-soluble, it is essential to keep them away from moisture. Storage becomes a problem in humid areas for dissolving microneedles, whereas storage conditions won’t affect derma rollers as their needles are stainless steel. In conditions where cream and microneedle patch has to be applied simultaneously, attachment of microneedle patch becomes difficult because of the left out the residue of cream (Hong et al. 2018). As the derma roller has to be rolled and not attached, it would be advantageous over the microneedle patch.

### Regulatory concerns in the use of derma rollers

The F.D.A. claims that dermarollers can be classified under section 513(f)(2) of the FD&C Act, also known as the De Novo classification system. This procedure provides a path to class I or class II classification for low-moderate risk devices for which general and unique controls or general controls provide a guarantee of efficacy and safety but for which there is no legally marketed predicate product. If a De Novo application for a microneedling device is approved, the particular product and system type would be listed as Class I or Class II and maybe subsequently marketed and act as a predicate for future products.

To facilitate a review, the De Novo request should provide all the information necessary to demonstrate a guarantee of the safety and efficacy of the system. The De Novo application will define the benefits and risks of the system and the general controls or general and special controls required to ensure safety, such as mitigation of any identified health risks and effectiveness. Data should be presented, showing that general controls or general and special controls endorse class I or class II classification. Based on the information currently available, the F.D.A. considers that risks associated with microneedling devices include infection, nerve and blood vessel disruption, user-to-user disease transmission, scar formation, hyperpigmentation, skin inflammation, allergic reactions, and skin irritation. Depending on the technical characteristics and intended uses of the specific microneedling tool, additional risks can be established (Guidance 2017).

## Conclusion and the way forward

Derma rollers have moved a long way from the traditional cosmetic applications to more complex transdermal drug delivery. Current data indicate that epidermal preservation is beneficial in aesthetic situations as the derma roller facilitates the development of collagen and elastin. Extensive studies are underway to examine responses to skin repair at a cellular level. In particular, its effectiveness, safety, and accessibility make the derma roller a successful therapeutic option. With many exciting studies published over the past few years, the use of derma roller technologies to facilitate the efficient delivery of pharmaceuticals is rapidly advancing. Derma roller results in disruption of the stratum corneum cells, which weakened the main barrier of the skin, causing an increase in the absorption of applied compounds. It is necessary to remember that most derma roller comparative studies are case reports, case series, or minor randomized controlled studies. Before microneedling is accepted for extensive use in patient care, there is much work to be done, but novel models address these issues. It is expected that, with the advancement in dermal and transdermal technology, derma roller will be approved clinically in the near future.

## Data Availability

All the data and material referred to in the manuscript is available in the public domain. Appropriate citations have been provided to the referred work of literature.
